# A novel computational model ITHCS for enhanced prognostic risk stratification in ESCC by correcting for intratumor heterogeneity

**DOI:** 10.1093/bib/bbae631

**Published:** 2024-12-17

**Authors:** Tong Lu, Wei Guo, Wei Guo, Wangyang Meng, Tianyi Han, Zizhen Guo, Chengqiang Li, Shugeng Gao, Youqiong Ye, Hecheng Li

**Affiliations:** Department of Thoracic Surgery, Ruijin Hospital, Shanghai Jiao Tong University School of Medicine, 197 Ruijin 2nd Road, Huangpu District, Shanghai 200025, China; Shanghai Institute of Immunology, State Key Laboratory of Oncogenes and Related Genes, Department of Immunology and Microbiology, Shanghai Jiao Tong University School of Medicine, 227 Chongqing South Road, Huangpu District, Shanghai 200025, China; Department of Thoracic Surgery, Ruijin Hospital, Shanghai Jiao Tong University School of Medicine, 197 Ruijin 2nd Road, Huangpu District, Shanghai 200025, China; Department of Thoracic Surgery, National Cancer Center/National Clinical Research Center for Cancer/Cancer Hospital, Chinese Academy of Medical Sciences, Peking Union Medical College, 17 Panjiayuan Nanli, Chaoyang District, Beijing 100021, China; Department of Thoracic Surgery, Ruijin Hospital, Shanghai Jiao Tong University School of Medicine, 197 Ruijin 2nd Road, Huangpu District, Shanghai 200025, China; Shanghai Institute of Immunology, State Key Laboratory of Oncogenes and Related Genes, Department of Immunology and Microbiology, Shanghai Jiao Tong University School of Medicine, 227 Chongqing South Road, Huangpu District, Shanghai 200025, China; Shanghai Institute of Immunology, State Key Laboratory of Oncogenes and Related Genes, Department of Immunology and Microbiology, Shanghai Jiao Tong University School of Medicine, 227 Chongqing South Road, Huangpu District, Shanghai 200025, China; Department of Neurosurgery, Center of Pituitary Tumor, Ruijin Hospital, Shanghai Jiao Tong University School of Medicine, 197 Ruijin 2nd Road, Huangpu District, Shanghai 200025, China; Department of Plastic and Reconstructive Surgery, Shanghai Ninth Peoples Hospital, Shanghai Jiao Tong University School of Medicine, 639 Zhizaoju Road, Huangpu District, Shanghai 200011, China; Department of Thoracic Surgery, Ruijin Hospital, Shanghai Jiao Tong University School of Medicine, 197 Ruijin 2nd Road, Huangpu District, Shanghai 200025, China; Department of Thoracic Surgery, National Cancer Center/National Clinical Research Center for Cancer/Cancer Hospital, Chinese Academy of Medical Sciences, Peking Union Medical College, 17 Panjiayuan Nanli, Chaoyang District, Beijing 100021, China; Shanghai Institute of Immunology, State Key Laboratory of Oncogenes and Related Genes, Department of Immunology and Microbiology, Shanghai Jiao Tong University School of Medicine, 227 Chongqing South Road, Huangpu District, Shanghai 200025, China; Department of Thoracic Surgery, Ruijin Hospital, Shanghai Jiao Tong University School of Medicine, 197 Ruijin 2nd Road, Huangpu District, Shanghai 200025, China

**Keywords:** esophageal squamous cell carcinoma, intra-tumor heterogeneity, sampling bias, machine learning, prognostic prediction

## Abstract

Intratumor heterogeneity significantly challenges the accuracy of existing prognostic models for esophageal squamous cell carcinoma (ESCC) by introducing biases related to the varied genetic and molecular landscapes within tumors. Traditional models, relying on single-sample, single-region bulk RNA sequencing, fall short of capturing the complexity of intratumor heterogeneity. To fill this gap, we developed a computational model for intratumor heterogeneity corrected signature (ITHCS) by employing both multiregion bulk and single-cell RNA sequencing to pinpoint genes that exhibit consistent expression patterns across different tumor regions but vary significantly among patients. Utilizing these genes, we applied multiple machine-learning algorithms for sophisticated feature selection and model construction. The ITHCS model significantly outperforms existing prognostic indicators in accuracy and generalizability, markedly reducing sampling biases caused by intratumor heterogeneity. This improvement is especially notable in the prognostic assessment of early-stage ESCC patients, where the model exhibits exceptional predictive power. Additionally, we found that the risk score based on ITHCS may be associated with epithelial-mesenchymal transition characteristics, indicating that high-risk patients may exhibit a diminished efficacy to immunotherapy.

## Introduction

Esophageal cancer ranks as the sixth leading cause of cancer-related mortality globally, accounting for 544 000 deaths in 2020, with >85% of these occurring in low- and middle-income countries, particularly in Central Asia and the Eastern Coast of Africa [1]. Among its types, esophageal squamous cell carcinoma (ESCC) is the most prevalent, constituting >90% of cases [1]. Despite advancements in ESCC treatment, the 5-year survival rate remains a mere 15.9%, with significant survival disparities [2]. The American Joint Committee on Cancer’s staging system, currently the foremost guideline for ESCC treatment, aids in classifying patient stages but lacks the precision needed for detailed stratification and accurate prognosis. This limitation underscores an urgent need for a more precise prognostic model that can facilitate accurate and personalized treatment decisions for ESCC patients. Addressing this gap is crucial for improving outcomes and equity in cancer care worldwide [3, 4].

In the last two decades, high-throughput sequencing technologies have significantly advanced our understanding of cancer biology, particularly in identifying RNA-based markers for cancer prognosis through transcriptomic data. Despite the development of several prognostic models for ESCC [5–8], their clinical application has been limited. This limitation may be attributed to a lack of high-quality, large-cohort ESCC datasets or inconsistencies in data quality, leading to unstable model performance. Furthermore, the inconsistency of data standards between different centers complicates data validation. In addition to these factors, a major challenge, as highlighted by previous research, is the inadequate consideration of tumor complexity, especially the role of tumor heterogeneity [9–11]. Tumor heterogeneity includes intertumoral (between different patients) and intratumoral (within the same tumor) heterogeneity. In our study, intratumoral heterogeneity refers to spatial genetic and molecular differences across different regions within a tumor. [12, 13]. Most prognostic models for ESCC have primarily focused on intertumor heterogeneity by analyzing samples from single tumor regions, neglecting intratumor diversity. This has led to models that may exhibit variability in performance across different patient populations and face challenges in replicability [9, 14, 15]. Recent studies using multiregion bulk genomic and single-cell transcriptomics have underscored the significance of intratumor heterogeneity in ESCC [16–19], indicating that integrating this variability could be key to developing more accurate prognostic models.

In this study, we aimed to analyze both inter- and intratumor heterogeneity in ESCC by integrating multidimensional transcriptomic data. This involved a critical evaluation of the efficacy of existing predictive models to elucidate the extent of inherent sampling bias. In this context, sampling bias refers to the variation in gene expression profiles that occurs when samples are taken from different regions of the same tumor. To address this, we devised a novel strategy to identify genes that are consistently expressed within individual tumors but vary significantly across different patients. Our approach seeks to establish a gene expression–based signature that effectively reduces the impact of intratumor heterogeneity, thereby enhancing the accuracy and reliability of prognostic evaluations for ESCC patients. By carefully designing our predictive model to overcome the challenges of clinical sample bias, we offer an improved prognostic risk assessment, providing a more robust tool for individuals diagnosed with ESCC.

## Material and methods

### Acquisition and processing of multiregion gene expression data

Multiregion sample data from patients with ESCC were procured from the GSE33426 [20]. This particular dataset is composed of microarray data pertaining to 71 samples, including 59 tumor regions and 12 normal esophageal regions, derived from 9 ESCC patients. Detailed sample collection information is depicted in Fig. S1. For probe annotation, we utilized the GPL571 [HG-U133A_2] platform annotation file corresponding to the Affymetrix Human Genome U133A 2.0 Array.

### Collection and analysis of single-region bulk RNA-seq and survival data

The current research integrated data from three distinct ESCC cohorts, encompassing clinical follow-up information. This compilation involved 432 ESCC samples from 430 patients and 182 normal samples, sourced from datasets GSE53625, TCGA-ESCC, and Zhang *et al*. (refer to Supplementary Table S1). The transcriptomic data for GSE53625 were derived from microarray, whereas those for TCGA-ESCC and Zhang *et al*. were obtained via RNA sequencing. Specifically, the GSE53625 dataset included 358 samples from 179 patients, featuring tumor samples along with demographic and clinical data such as age, gender, staging, and tumor grading. Probe were annotated using the GPL18109 platform file for the Agilent-038314 CBC *Homo sapiens* lncRNA + mRNA microarray V2.0. For the TCGA-ESCC cohort, transcriptomic data for 95 ESCC specimens and 3 normal specimens from 93 patients were acquired from TCGA Genomic Data Commons (GDC) (https://portal.gdc.cancer.gov/repository). Transcripts per million (TPM) values were extracted for further analysis, focusing exclusively on primary tumor samples (TCGA code 01A), resulting in data for 93 primary ESCC tumors. Additional data, including somatic mutation profiles and clinical–pathological features with follow-up information for ESCC patients, were also retrieved from the GDC. Homologous recombination deficiency (HRD) and cell stemness scores were sourced from the TCGA PanCancer Atlas (https://pancanatlas.xenahubs.net). Another cohort, analyzed as per Zhang *et al*. [21], included 159 ESCC patients with accompanying clinical survival data and tumor staging, with the dataset comprising TPM values.

### Acquisition and processing of single-cell RNA data

Three cohorts of single-cell RNA sequencing for ESCC were incorporated: GSE196756 (encompassing 3 tumor and 3 normal tissues), GSE197677 (with 18 tumor and 12 normal tissues), and GSE160269 (comprising 60 tumor tissues) (Supplementary Table S2). From these datasets, 3 tumor specimens from GSE196756, 4 from GSE197677, and 60 from GSE160269 were meticulously extracted and subsequently processed to establish individual Seurat objects via the Seurat package [22]. We implemented a filtering criterion, excluding cells exhibiting >5% mitochondrial gene content. Further, cells that did not meet the established quality control parameters (nFeature_RNA < 6000, nFeature_RNA > 200) were eliminated, and the presence of doublets was addressed using the doubletFinder_v3 function. The SCTransform method facilitated the normalization of the dataset. We then selected the principal 15 components for significant statistical input in the uniform manifold approximation and projection (UMAP) analysis. The study involved annotating three distinct cell types, namely, epithelial cells, immune cells, and stromal cells, identified through specific marker genes: KRT14, KRT18, EPCAM, and SFN for epithelial cells; PTPRC and JCHAIN (B cells) for immune cells; and PECAM1, FN1, and VWF for stromal cells. The chosen marker genes are based on previously published literature [21, 23, 24]. We applied an entropy-based statistic, ROGUE, to quantify the heterogeneity within various cell subpopulations [25]. This approach enabled the evaluation of heterogeneity scores for subgroups of epithelial, stromal, and immune cells, with higher ROGUE scores indicating a lower degree of heterogeneity within each subgroup.

### Analytical approach to tumor heterogeneity and quadrant mapping

Intra- and intertumor heterogeneity were quantitatively analyzed using the multiregion gene expression data procured from the GSE33426 cohort. To quantify intratumor heterogeneity for each gene, we calculated the standard deviation (SD) of gene expression levels across different tumor regions. This methodology employs multiregion tumor sampling to evaluate heterogeneity within the tumor. In our analysis, we employed metrics such as SD, median absolute deviation (MAD), and coefficient of variation (CV) to measure gene expression heterogeneity, ultimately selecting SD as the most suitable metric for our study.

For assessing intertumor heterogeneity, we employed the methodology previously described by Luo *et al* [10]. A random tumor area was selected per patient, and the SD for each gene was calculated. This random selection process was repeated 10 times, and the mean value of these iterations was used to establish the intertumor heterogeneity score. A quadrant system for gene heterogeneity (Q1–Q4) was devised based on the mean scores of intra- and intertumor heterogeneity.

### Compilation of prognostic gene expression signatures

This investigation entailed an extensive collection of 13 prognostic risk models pertaining to ESCC, which have been previously elucidated in the literature [6, 8, 26–36]. Among these, we meticulously selected 13 models that were constructed using the Cox proportional hazards method. Each model was closely associated with a distinct predictive formula, as detailed in Supplementary Table S3. These models employed the formula for signature risk score as:


$$ {\sum}_{i=1}^n\Big(\left( coefficient\ of\ gene\ i\right)\times \left( expression\ value\ of\ gene\ i\right) $$

where “i” symbolizes the respective gene and “*n*” the total gene count. This formula, consistent across gene lists and coefficients, was applied across various expression matrices to ascertain the signature risk score for individual samples. In each of the 13 models, samples were stratified into high-risk or low-risk categories based on the median risk score cutoff values derived from each cohort.

For each tumor region, a risk score was computed. Patients with exclusively low-risk regions were categorized as “concordant low risk,” those with only high-risk regions as “concordant high risk,” and those with a combination as “discordant.” A model was regarded as exhibiting substantial sampling bias if the “discordant” classification was applicable to >50% of the patients.

### Prognostic gene selection based on heterogeneity quartiles

Utilizing the heterogeneity quartile gene sets, we conducted univariate Cox regression analysis for the Q1, Q2, Q3, Q4, and Q1–Q4 gene sets, with a primary focus on the Q4 gene set, which is distinguished by high intertumor heterogeneity and low intratumor heterogeneity. The univariate Cox analysis was performed using the survival package in R (https://CRAN.R-project.org/package=survival), applying a *P*-value <.05 for gene selection. The GSE53625 dataset served as the primary training set for this screening.

### Construction and validation of an intratumor heterogeneity prognostic model

A least absolute shrinkage and selection operator (LASSO) regression analysis of the univariate Cox results was conducted using the glmnet package [37], refining the selection to genes most indicative of prognostic relevance.

To achieve a model with enhanced precision and robustness, we integrated a suite of nine machine learning algorithms with the following parameters: random survival forests (RSFs) (ntree = 1000), elastic net (Enet) (alpha ranging from 0.1 to 0.9), ridge regression (α = 0), stepwise Cox regression (StepCox) (direction = both, forward, backward), CoxBoost (maxstepno = 500, K = 10, type = “verweij”), partial least squares regression for Cox models (plsRcox) (nt = 5), supervised principal components (SuperPC) (type = “survival”, s0.perc = 0.5, n.threshold = 20, n.fold = 10, n.components = 3, min.features = 5), gradient boosting machine (GBM) (n.trees = 10 000, interaction.depth = 3, n.minobsinnode = 10, shrinkage = 0.001), and survival–support vector machine (Survival-SVM) (gamma.mu = 1). Based on the genes selected by the univariate and LASSO analysis, models were trained using these algorithms and subjected to rigorous internal validation through 5-fold cross-validation [set.seed(123)]. Subsequently, external validation was performed using the TCGA-ESCC and Zhang *et al*. [21] datasets. Harrell’s concordance index (C-index) was computed across all datasets to determine the optimal model. Additionally, we evaluated the temporal accuracy of the models using the timeROC R package [38], calculating the area under the curve (AUC) over time for both the training and test sets.

### Analytical comparison of high-risk versus low-risk categories

This study stratified patients from datasets GSE53625, TCGA-ESCC, and Zhang *et al*. into high-risk and low-risk groups based on median risk scores. Differential gene expression analysis was conducted using the limma package [39], accommodating data types such as microarray (GSE53625) and RNA-seq TPM values (TCGA-ESCC and Zhang *et al*.). Gene set enrichment analysis (GSEA) [40] was then employed to conduct enrichment analysis on these stratified groups using the comprehensive “h.all.v2023.2.Hs.symbols.gmt” Hallmark gene set. Additionally, the IOBR package [41] was deployed for the quantitative assessment of immune cell infiltration and the characterization of various biological signatures in the aforementioned datasets.

In an effort to elucidate the differential response to immune therapy among high-risk and low-risk patients, this study integrated data from six pertinent immune therapy datasets: IMvigor210 [42], GSE213331 [43], GSE91061 [44], GSE115821 [45], GSE135222 [46], and GSE126044 [47] (Supplementary Table S4). The comparative analysis focused on the risk score discrepancies between patients classified as responsive (pathological complete response [pCR]) and nonresponsive (nonpathological complete response [npCR]) to the treatment.

### Spatial transcriptomic analysis

Spatial transcriptomics data acquisition and processing methodologies adhered to previously established protocols [48]. The Seurat software suite was employed for downstream analysis, including data normalization using the SCTransform function. The computational tool AddModuleScore was applied to determine the scores for 13 specific signatures and intratumor heterogeneity corrected signature (ITHCS), subsequently analyzing their SDs to evaluate spatial heterogeneity. To assess the epithelial and stromal areas at the spatial level, we scored the spot using the ESTIMATE algorithm [49].

### Assessing tumor heterogeneity across different prognostic signatures in esophageal squamous cell carcinoma

In the ESCC bulk RNA-seq datasets GSE33426, GSE53625, TCGA-ESCC, and Zhang *et al*., we applied the previously mentioned risk scoring formula to calculate the risk scores for the ITHCS and other reported ESCC prognostic signatures (a total of 14 prognostic signatures). Then, we used variance analysis to compare differences in intratumor and intertumor heterogeneity among them. In the single-cell datasets GSE196756, GSE197677, and GSE160269 and the spatial transcriptomics dataset by Guo *et al*. [48], we employed the AddModuleScore function, based on the genes of 14 prognostic signatures, to calculate signature scores for cells. Variance analysis was applied to compare differences in intratumoral heterogeneity at the single-cell and spatial levels.

### Statistical analysis

Comprehensive statistical analyses, including data visualization, were conducted using R software version 4.3.2 (https://r-project.org/). Correlations between continuous variables were determined using Pearson’s correlation in cases of normal distribution and Spearman’s correlation for non-normally distributed data. Comparative analyses of continuous variables were executed employing Student’s *t*-test or Wilcoxon rank-sum test, while categorical variables in contingency tables were analyzed using Fisher’s exact test. A two-tailed *P*-value threshold of <.05 was set for establishing statistical significance.

## Results

### The impact of sampling bias on esophageal squamous cell carcinoma prognostic signature resulting from intratumor heterogeneity

**Figure 1:**
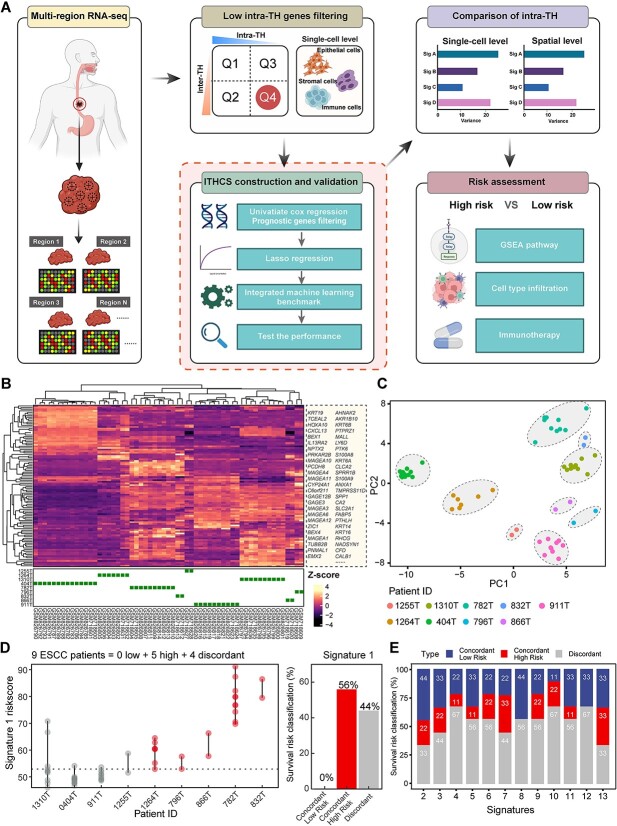
Impact of intratumor heterogeneity on sampling bias. (A) Comprehensive overview of the study design. (B) The heatmap (top) illustrates unsupervised hierarchical clustering based on the 100 most variably expressed genes within the GSE33426 cohort. The *x*-axis represents the highly variable genes, and the *y*-axis represents the tumor samples. The heatmap (bottom) presents the sample distribution for each patient with ESCC. The *x*-axis represents the ESCC patient IDs, and the *y*-axis represents the tumor samples. (C) PCA using highly variable genes in the GSE33426 dataset. (D) Tumor samples from individual regions are represented by points (left). The median risk score across these samples is denoted by a horizontal dashed line. The accompanying bar chart (right) displays the proportion of patients classified into consistent low-risk, high-risk, and inconsistent-risk categories. (E) Bar chart shows the percentage of patients in the GSE33426 cohort categorized into low, high, and disconcordant risk groups based on an analysis of 12 established signatures. ESCC, esophageal squamous cell carcinoma; PCA, principal component analysis.

The workflow of this study is depicted in Fig. 1A, began with unsupervised stratified clustering of highly variable genes across 59 tumor regions from the multiregion GSE33426 cohort, revealing significant clustering consistency within different regions of the same tumor (Fig. 1B). This finding suggests that RNA intertumor heterogeneity may be more pronounced than intratumor heterogeneity. Moreover, a comprehensive dimensionality reduction of the entire transcriptomic profile distinctly unveiled the RNA intratumor heterogeneity across various regions of the same ESCC. The reliance on molecular characteristics obtained from a single region may lead to biases in clinical strategies, encompassing biopsy approaches, molecular pathological diagnosis, targeted therapy, and prognostic prediction. To assess the impact of RNA intratumor heterogeneity on the performance of molecular biomarkers, we applied 13 established ESCC prognostic models in the GSE33426 cohort, assessing for patient risk bias. Initially, we evaluated the performance of a recently developed RNA-Seq-based prognostic signature (Signature 1) by Zhao *et al*. [26]. Using the same risk scoring method as the original study, tumor regions were classified as high-risk or low-risk. It was observed that 44% (4/9) of the patients exhibited discordant categorization (Fig. 1D). The remaining 12 predictive models demonstrated an average inconsistency rate of 52.9% (ranging from 33% to 67%) (Fig. 1E). These findings highlight that known prognostic models, which overlook intra-tumor heterogeneity, are susceptible to sample bias, reducing their effectiveness and reliability in independent cohorts. This limitation underscores the need for new strategies in the clinical application of ESCC prognostic models.

### Identification of genes with low intratumor heterogeneity and high intertumor heterogeneity

**Figure 2:**
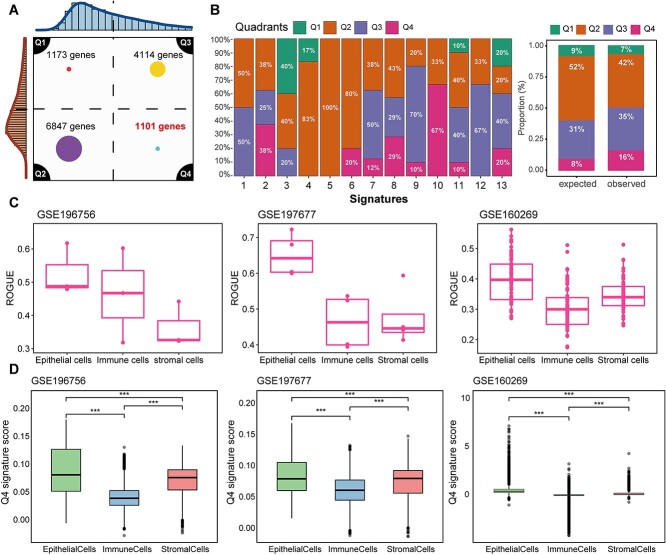
Screening for low intratumor heterogeneity genes. (A) RNA heterogeneity quadrant chart based on the GSE33426 multiregion ESCC cohort. The *x*-axis represents intertumor heterogeneity, while the *y*-axis denotes intratumor heterogeneity. (B) Proportion of genes from published ESCC prognostic models within quadrants Q1–Q4 (left). Percentage of expected versus observed genes in each RNA heterogeneity quadrant. (C) ROGUE scores for epithelial cells, immune cells, and stromal cells in ESCC single-cell RNA cohorts GSE196756, GSE197677, and GSE160269. (D) Boxplot comparison of Q4 quadrant gene feature scores among epithelial cells, immune cells, and stromal cells in the GSE196756, GSE197677, and GSE160269 cohorts. Statistical analysis was performed using the Kruskal–Wallis test followed by Dunn’s test. ***, *P* < .001. ESCC, esophageal squamous cell carcinoma.

The design of biomarkers can be optimized by minimizing sampling bias, which is induced by intratumor heterogeneity while maximizing the ability to distinguish between different tumors (intertumor heterogeneity). This approach aims to identify prognostic models that, compared to existing prognostic markers, exhibit higher reproducibility and clinical utility. Utilizing the GSE33426 dataset, we derived scores for intratumor and intertumor heterogeneity of genes. Based on these heterogeneity indices, genes were classified into high or low groups, resulting in an RNA heterogeneity quadrant chart (Fig. 2A). The quadrants included genes with low inter- and high intraheterogeneity (Q1 = 1173 genes), low inter- and low intraheterogeneity (Q2 = 6847 genes), high inter- and high intraheterogeneity (Q3 = 4114 genes), and high inter- and low intraheterogeneity (Q4 = 1107 genes) (Supplementary Table S5). Genes in Q4 met the desired criteria: exhibiting homogenous expression within tumors, thus limiting sampling bias, but showing high variability between tumors, potentially offering valuable information for patient stratification. Correlation analysis between standard deviation, MAD, and CV indicated a significant positive correlation among them (SD and MAD *r* = 0.95, *P* < 2.2e-16; SD-CV *r* = 0.685, *P* < 2.2e-16; MAD-CV *r* = 0.65, *P* < 2.2e-16) (Fig. S3A). This suggests the absence of significant outliers in the GSE33426 dataset, with stable mean values and SD being an effective measure of data dispersion. The RNA heterogeneity quadrant chart showed that genes in Q4 constituted 8.3% (1101/13 235) of all expressed genes, but comprised 16% of genes identified from 13 previously published prognostic features, indicating a 2-fold enrichment (Fig. 2B). This suggests that previous studies tended to select Q4 genes even in the absence of RNA-ITH information.

In previous research, Dunne *et al*. inferred from the bulk data of colorectal cancer that gene signatures based on inherent epithelial cell gene expression could significantly enhance patient stratification accuracy in colorectal cancer, compared to stroma-dependent signatures [50]. From a single-cell perspective, we initially distinguished cell subgroups in single-cell datasets GSE196756, GSE197677, and GSE160269, categorizing them into epithelial, immune, and stromal cell groups based on marker genes (Fig. S2). Calculating ROGUE scores for different subgroups revealed that the heterogeneity of the epithelial cell subgroup in all ESCC single-cell datasets was significantly lower than that of the immune and stromal cell subgroups (Fig. 2C). Further, using the addmodulescore algorithm, we assessed the scores of genes in the Q4 quadrant across different cell subtypes. The results indicated that the epithelial cell subgroup scored significantly higher than the immune and stromal cells in the single-cell datasets (Fig. 2D). Additionally, we observed that the dispersion of gene expression in the Q4 quadrant was significantly lower in the single-cell datasets compared to quadrants Q1–Q3 (Fig. S3B). These findings suggest that genes in the Q4 quadrant are characterized by low intratumor heterogeneity and are likely more derived from inherent epithelial cell genes.

### Construct an esophageal squamous cell carcinoma prognostic model that enhances prediction efficacy by minimizing intratumor heterogeneity

**Figure 3:**
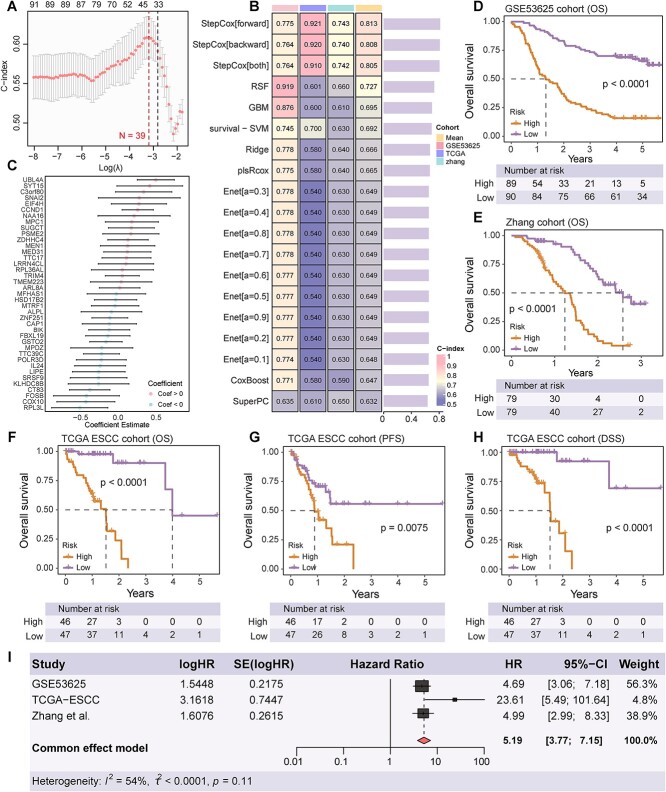
Development of the ITHCS. (A) Lasso regression’s 5-fold cross-validation AUC graph under varying lambda parameters. (B) C-index calculated after integrating 9 machine learning algorithms, using GSE53625 as the training cohort and TCGA-ESCC and Zhang *et al*. as validation cohorts. (C) Coefficient estimates for selected genes in the model are shown, with each dot representing the estimated coefficient for a gene. Error bars indicate the 95% confidence intervals for these coefficients. (D–F) Patient OS analysis using the ITHCS risk score in the GSE53625 cohort (training set), Zhang *et al*. (external test set), and TCGA-ESCC (external test set). Patients in each dataset were divided into high-risk and low-risk groups based on median risk score. In all three datasets, patients in the high-risk group exhibited significantly poorer prognosis compared to those in the low-risk group. (G, H) In the TCGA-ESCC cohort, patient risk scores calculated using ITHCS were used to assess PFS and DSS. The results indicated that patients in the high-risk group had poorer PFS and DSS outcomes compared to those in the low-risk group. (I) A combined analysis based on the GSE53625, TCGA-ESCC, and Zhang *et al*. cohorts revealed that the ITHCS risk score consistently served as a risk factor associated with poorer patient prognosis, with no heterogeneity differences observed across the three datasets (*P* = .11). ITHCS, intratumor heterogeneity corrected signature; C-index, concordance index; OS, overall survival; PFS, progression-free survival; DSS, disease-specific survival.

The strategy for model development is illustrated in Fig. S4. To construct an ESCC prognostic model that minimally affects intratumor heterogeneity, we utilized the GSE53625 dataset as a training set. This dataset, with its larger sample size compared to the TCGA-ESCC and Zhang *et al*. cohorts, provided a more robust foundation for model training. Within the GSE53625 dataset, an initial univariate Cox analysis was conducted on genes in the Q4 quadrant to preliminarily screen for genes correlated with prognosis. The results identified several genes that conformed to the criteria of the univariate Cox analysis (Supplementary Table S6). Subsequently, we employed the lasso algorithm to refine the selection of feature genes, eliminating redundancy and identifying the most promising prognostic markers. The lasso analysis determined the optimal lambda value (*N* = 39) when the C-index was at its highest (Fig. 3A). Utilizing GSE53625 as the training set and TCGA-ESCC and Zhang *et al*. as independent external validation cohorts, we found that among all algorithms, StepCox[forward] exhibited the highest average C-index (Average C-index = 0.813). While RSF and GBM showed superior performance in the training set compared to StepCox, their efficacy diminished in the external test sets, likely indicating model overfitting (Fig. 3B). Based on the results from StepCox, we noted that out of the 39 genes, 20 had a coefficient <0, suggesting their potential as protective factors, while 19 with a coefficient >0, indicating possible risk factors (Fig. 3C, Supplementary Table S7). Risk scores for each patient in both the training and testing sets were calculated based on the expression of these genes and their weighted regression coefficients. Patients were then stratified into high-risk and low-risk groups according to the median value. As depicted in Fig. 3D–F, in the GSE53625 training data and the two additional test datasets, the overall survival rate (OS) of patients in the high-risk group was significantly lower than those in the low-risk group (*P* < .0001). Furthermore, in comparisons of progression-free survival (PFS) and disease-specific survival (DSS) within the TCGA-ESCC dataset, a poorer prognosis was observed for patients in the high-risk group compared to those in the low-risk group (*P* < .05) (Fig. 3G and H). Across the three datasets, the predictive model exhibited hazard ratios (HRs) significantly >1, with no substantial heterogeneity observed in these studies (*I*^2^ = 54%, *P* = .11). To validate the accuracy of the Q4 gene set model, we compared it against models constructed from other quartile gene sets, including Q1, Q2, Q3, and Q1–Q4. Following univariate Cox regression and lasso selection, the final number of genes in each set was as follows: Q1 = 33, Q2 = 39, Q3 = 43, and Q1–Q4 = 55 (Fig. S5A). The final model demonstrated that the Q4 gene set achieved the highest accuracy (Fig. S5B). These findings suggest that the StepCox predictive model, encompassing 39 genes, performs comparably or better than other models in terms of accuracy and stability, and effectively distinguishes survival differences among patients.

**Figure 4:**
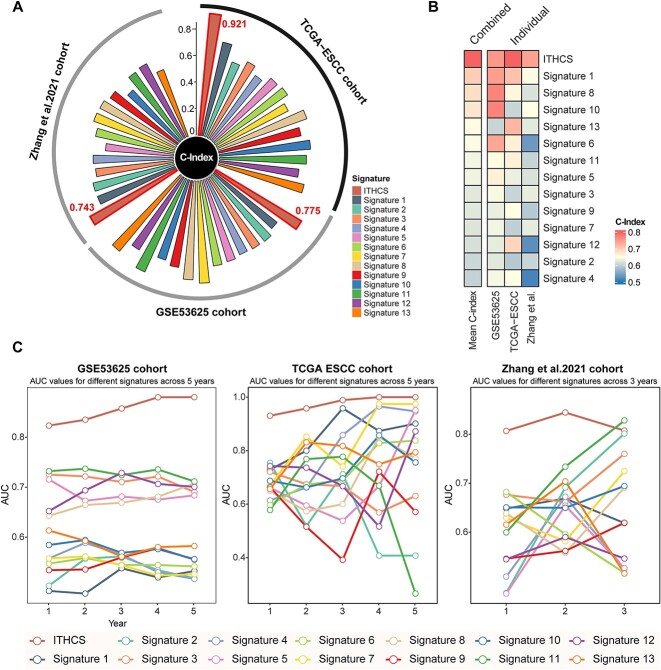
Comparative prognostic accuracy of ITHCS and other models. (A, B) Circular plot depicting the comparison of the C-index between ITHCS and 13 other signatures across three datasets. The vertical axis represents the C-index (left). Accompanying heatmaps illustrate the C-index comparison between ITHCS and the 13 other signatures across the three datasets, highlighting that ITHCS consistently achieves a higher average C-index than other models. (C) Comparative analysis of the AUC values between ITHCS and other signatures over different years. The left section presents data from the GSE53625 cohort, the middle from the TCGA-ESCC cohort, and the right from the Zhang *et al*. cohort. ITHCS, intratumor heterogeneity corrected signature; C-index, concordance index; AUC, area under the curve.

### The intratumor heterogeneity corrected signature outperforms other esophageal squamous cell carcinoma prognostic models

Given the previously reported prognostic signatures demonstrating good performance in predicting the prognosis of ESCC patients, we concurrently evaluated the discriminative and prognostic accuracy of 13 established multigene signatures alongside ITHCS. As shown in Fig. 4A and B, the C-index of ITHCS surpassed that of the previously reported 13 models in the overall dataset (most of which were primarily developed based on TCGA-ESCC training [9/13]). The timeROC curves revealed that, in the GSE53625 and TCGA-ESCC datasets, the AUC values of ITHCS were higher than those of the remaining 13 models, with a notably superior AUC in GSE53625 compared to others. Although in the Zhang *et al*. cohort, the AUC of ITHCS in the third year was slightly lower than that of Signature 11, the comprehensive results across the three datasets demonstrated a superior predictive accuracy of the ITHCS model over previously reported models.

**Figure 5:**
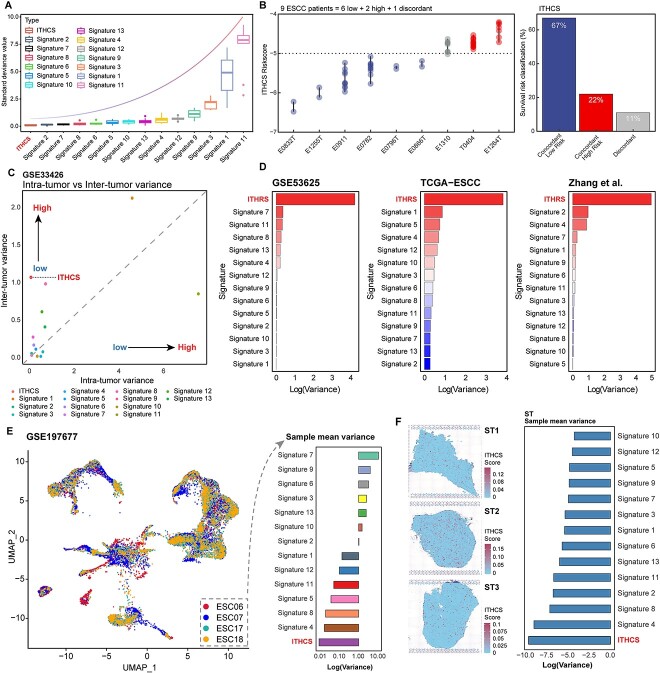
Tumor heterogeneity assessment in different transcriptomic levels using ITHCS. (A) Box plots in the GSE33426 cohort, comparing the standard deviation of ITHCS risk scores with those of 13 other models for patients’ multiregion samples. (B) In the GSE33426 cohort, ITHCS evaluation (left) is illustrated by a bar graph, demonstrating the percentage of patients categorized into low-consistency risk, high-consistency risk, and inconsistent risk groups based on ITHCS risk scoring. (C) Comparison of intratumor and intertumor heterogeneity among 14 signatures in the GSE33426 cohort. The *x*-axis represents intratumor heterogeneity, while the *y*-axis represents intertumor heterogeneity. (E) On the left, the UMAP plot of the GSE197677 dataset shows the distribution of single cells within the samples. On the right, the bar chart presents the average variance of the 14 signature scores for the samples. Lower variance indicates reduced intratumor heterogeneity, with the *x*-axis representing log2-transformed variance values. (F) In the spatial transcriptomics cohort, a comparison of mean risk deviation between ITHCS and 13 other models in three tumor samples, with the *x*-axis showing the SD post-log2 transformation. The right panel presents a slice diagram illustrating the distribution of the ITHCS signature in spots. ITHCS, intratumor heterogeneity corrected signature; UMAP, uniform manifold approximation and projection.

The ITHCS not only demonstrated the highest accuracy but also exhibited characteristics of reduced intratumor heterogeneity. Compared to other models, ITHCS predicted the lowest median risk deviation in multiregion samples from the same patient (Fig. 5A). Furthermore, only 11% (1/9) of the ESCC patients showed inconsistent risk classification using ITHCS, outperforming the previously reported 13 ESCC predictive models (Fig. 5B). When comparing the differences in intratumor and intertumor heterogeneity across the previously mentioned 14 signatures, it was found that ITCHS not only demonstrates the lowest level of intratumor heterogeneity but also preserves a comparatively high level of intertumor heterogeneity, achieving the second-highest ranking in this respect among the 14 signatures (Fig. 5C). In addition, we validated the variance of the ITHCS risk score compared to the 13 other models’ risk scores across three bulk datasets. A larger variance indicates greater heterogeneity among different samples, which means higher inter-tumor heterogeneity and better patient differentiation. The results showed that the ITHCS model had a significantly higher risk score variance than the other 13 models, indicating a more distinct patient stratification capability (Fig. 5D). These findings suggest that ITHCS effectively mitigates sampling bias in bulk analysis, facilitating its application in single biopsy–based patient risk stratification.

At the single-cell level, we assessed the heterogeneity of risk deviation between ITHCS and other models. In the GSE197677 dataset, among four tumor samples, ITHCS exhibited the lowest risk deviation, indicating its low heterogeneity at the single-cell level (Fig. 5G and H). In the GSE196756 dataset, ITHCS’s average risk deviation was second to last, slightly higher than Signature 4 (Fig. S6A and B). In the GSE160269 dataset, ITHCS showed the lowest average risk deviation (Fig. S6C and D). Given that GSE160269 contains single-cell data from 60 samples, these results are more objectively robust. Hence, at the single-cell level, ITHCS also presents the lowest intratumor heterogeneity. Additionally, we evaluated the risk deviation of ITHCS and other models at the spatial level. Although previous research differentiated epithelial and stromal regions, in this study, we reassessed the epithelial and stromal areas of the sections using the ESTIMATE algorithm, as shown in Fig. S7A–C. This outcome was nearly consistent with our previous regional divisions [48]. Subsequently, we assessed the risk distribution of ITHCS scores versus those from other models within three ESCC samples. For each sample, we calculated the risk scores across all spots for the 14 signatures and then determined each signature’s variance. The average variance across these samples was used to evaluate the overall heterogeneity differences between the signatures. Our findings revealed that ITHCS displayed the lowest level of spatial heterogeneity in the three ESCC samples (Fig. 5I). These results from multidimensional transcriptomic data analyses suggest that ITHCS not only effectively mitigates the impact of intratumor heterogeneity but also retains the distinctive characteristics of intertumor heterogeneity.

### Intratumor heterogeneity corrected signature as an independent prognostic factor with distinct stratification for early-stage esophageal squamous cell carcinoma patients

Exploring the correlation of ITHCS with clinical features in bulk datasets, we observed that a higher proportion of patients in the high-risk group were in advanced stages (GSE53625: 53% versus 40%; TCGA-ESCC: 41% versus 23%; Zhang *et al*.: 56% versus 41%, Fig. S8A). When comparing ITHCS with other clinical characteristics such as age, gender, and drinking habits, no significant differences were found in the GSE53625 and TCGA-ESCC datasets (Fig. S8B and C). A significant mutant gene (SMG) analysis was conducted in the TCGA-ESCC cohort. The mutation spectrum revealed TP53 (87%) and TTN (30%) as the two most common mutant genes in ESCC (Fig. S8D). Overall, patients in the high-risk and low-risk groups did not exhibit significant differences in mutant genes. Comparing high-risk groups with low-risk groups, no significant differences were found in levels of TMB and MSI (TMB: *P* = .70, MSI: *P* = .52) (Fig. S8E). Additionally, no significant correlations were observed between genomic instability-related features such as HRD, loss of heterozygosity (LOH), and the ITHCS score (Fig. S8F), nor was a significant correlation observed between stemness score and ITHCS score (Fig. S8G).

**Figure 6:**
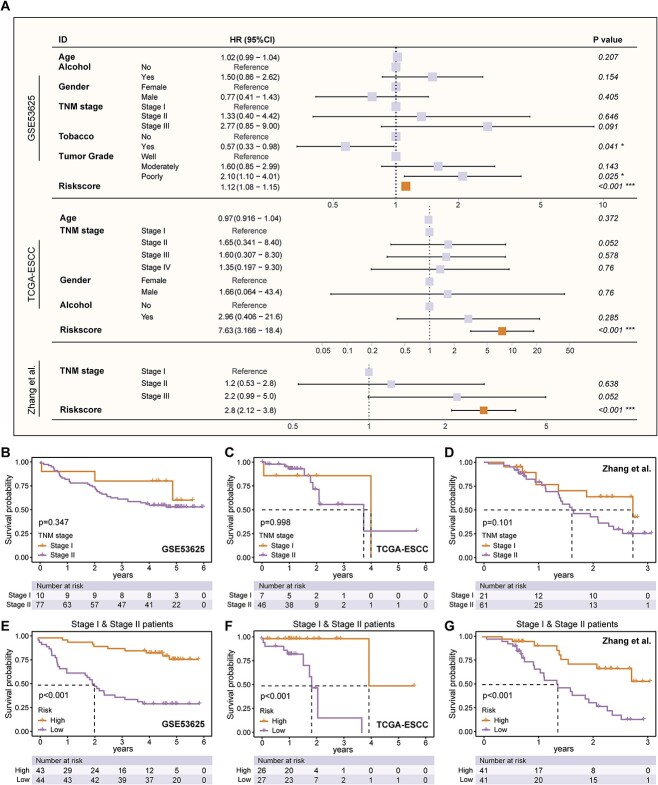
ITHCS demonstrates superior performance in early-stage risk stratification of ESCC. (A) Multivariate Cox regression analysis was conducted on the GSE53625, TCGA-ESCC, and Zhang *et al*. cohorts. ITHCS risk scores were identified as independent prognostic factors in all three datasets, as indicated by orange labels. (B–D) Kaplan–Meier analysis for Stage I and Stage II patients was performed in the GSE53625, TCGA-ESCC, and Zhang *et al*. cohorts. The results indicate that the prognosis differences between Stage I and Stage II patients in these datasets are not significant. (E–G) The ITHCS risk scores were applied for risk stratification of Stage I and II patients in the GSE53625, TCGA-ESCC, and Zhang *et al*. cohorts. Findings reveal that the ITHCS risk scoring effectively distinguishes between high-risk and low-risk patient groups in all three datasets. ITHCS, intratumor heterogeneity corrected signature; KM, Kaplan–Meier.

After multivariate Cox analysis of clinical features such as age, gender, TNM staging, and ITHCS scores in the three bulk datasets, ITHCS remained significantly associated with prognosis, indicating its role as an independent prognostic factor (Fig. 6A). Although the TNM staging system is significant in monitoring the prognosis of ESCC patients, some patients in Stages I and II still experience rapid recurrence or metastasis leading to death postsurgery. This may reflect the limitations of the TNM staging system in the risk stratification of early-stage patients. Thus, we focused on the capability of ITHCS for risk stratification in early-stage patients. As shown in Fig. 6B–D, TNM staging has limitations in differentiating between stage I and II ESCC patients, with the current TNM staging system showing limited prognostic differentiation in all three datasets. Subsequently, we grouped patients in Stages I and II and stratified them into high- and low-risk groups based on their risk scores. The results demonstrated that risk stratification based on ITHCS significantly differentiated early-stage patients (Fig. 6E–G). Compared with other models, while Signatures 2, 4, 7, 9, and 13 were observed to stratify early-stage risk in two datasets, only ITHCS showed significant statistical significance across all three datasets (Fig. S9A–C). These findings indicate that the ITHCS score is not only an independent prognostic factor but also provides a more effective assessment of survival in early-stage patients.

### Intratumor heterogeneity corrected signature associated with epithelial–mesenchymal transition and immune therapy resistance

**Figure 7:**
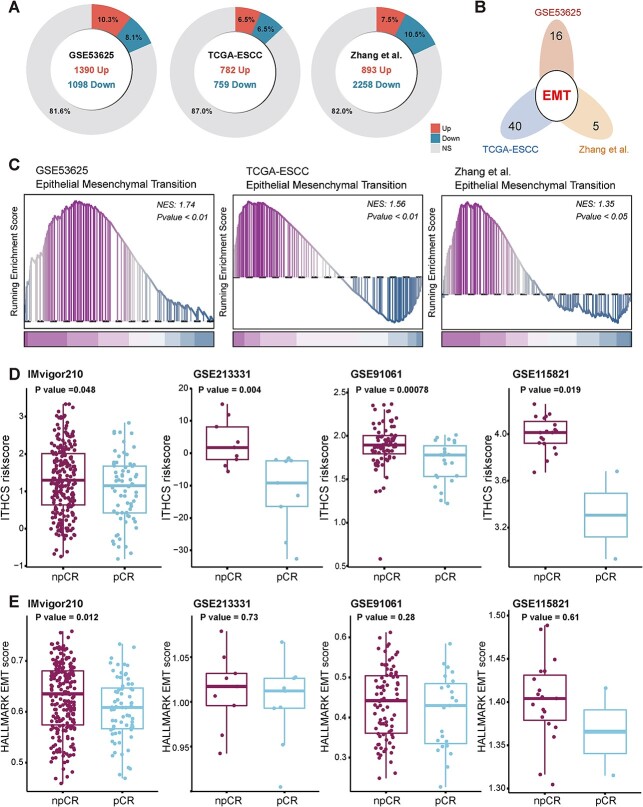
Potential biological differences between high-risk and low-risk patients as identified by ITHCS. (A) Proportion and number of differentially expressed genes in high-risk and low-risk groups across GSE53625, TCGA-ESCC, and Zhang *et al*. cohorts. (B) Intersection results of GSEA in GSE53625, TCGA-ESCC, and Zhang *et al*. (C) Enrichment results of the HALLMARK EMT pathway in GSE53625, TCGA-ESCC, and Zhang *et al*. cohorts. (D) Differences in npCR and pCR between high-risk and low-risk groups in immune therapy datasets IMvigor210, GSE213331, GSE91061, and GSE115821, with significantly higher risk scores observed in the high-risk group. (D) Differences in ITHCS risk scores between npCR and pCR in immunotherapy datasets IMvigor210, GSE213331, GSE91061, and GSE115821. Risk scores for npCR are notably higher than those in the low-risk group. (E) Differences in EMT scores between npCR and pCR in immunotherapy datasets. In the IMvigor210 dataset, npCR scores significantly exceed those of pCR, with no significant statistical differences observed in other datasets. GSEA, gene set enrichment analysis; EMT, epithelial–mesenchymal transition; pCR, pathological complete response; npCR, nonpathological complete response.

Based on the median values of patients in the GSE53625, TCGA-ESCC, and Zhang *et al*. cohorts, patients were classified into high-risk and low-risk groups. In the GSE53625 dataset, it was observed that 1390 genes were significantly upregulated in the high-risk group (accounting for 10.3% of all genes), while 1098 genes were significantly elevated in the low-risk group (representing 8.1% of all genes). In TCGA-ESCC, 782 genes were upregulated in the high-risk group (6.5% of all genes), and 759 genes were upregulated in the low-risk group (6.5% of all genes). In Zhang *et al*., 893 genes were upregulated in the high-risk group (7.5% of all genes), with 2258 genes upregulated in the low-risk group (10.5% of all genes) (Fig. 7A). Subsequently, the GSEA results from the HALLMARK gene set indicated that GSE53625, TCGA-ESCC, and Zhang *et al*. were enriched in 23, 39, and 5 significantly different HALLMARK features, respectively (Table S8). Notably, only the EMT feature was commonly enriched across all three datasets (Fig. 7B). The GSEA results revealed significant enrichment of the EMT pathway in high-risk group patients across all three datasets, with respective normalized enrichment scores (NESs) of 1.74 (GSE53625), 1.56 (TCGA-ESCC), and 1.35 (Zhang *et al*.) (Fig. 7C).

Immune cell infiltration, especially the infiltration of CD8+ T cells, is often a crucial determinant in tumor prognosis. It was hypothesized that patients in the high-risk group might exhibit lower levels of immune cell infiltration. To assess this, we employed the CIBERSORT to evaluate differences in immune cell infiltration between high-risk and low-risk groups. However, there were no significant differences observed in immune cell infiltration between patients in the high-risk group and those in the low-risk group (Fig. S10A). Further comparisons of the expression of CD8 effector and immune checkpoint–related genes revealed no notable statistical differences between the high and low-risk groups (Fig. S10B and C).

A previous study has indicated a correlation between high EMT features and resistance to tumor immune therapy [51]. We synthesized six immune therapy datasets and calculated the risk scores for each patient using the ITHCS formula. Results indicated that in the IMvigor210, GSE213331, GSE91061, and GSE115821 datasets, patients with npCR had significantly higher ITHCS risk scores than those with pCR (Fig. 7D). Although no significant statistical differences were observed in the remaining two datasets, npCR patients exhibited higher risk scores than pCR patients (Fig. S11A and B). Additionally, we compared the EMT scores between patients with pCR and npCR across these immunotherapy datasets. Notably, within the IMvigor210 dataset, npCR patients had significantly higher EMT scores than pCR patients. While other datasets did not show a statistical difference in EMT scores, the general trend suggested that npCR patients tended to have higher EMT scores than their pCR counterparts (Fig. 7E, Fig. S11C and D). Based on these findings, we propose that the defining genes in ITHCS largely derive from those associated with low intratumor heterogeneity and are closely linked to characteristics of epithelial or stromal cells. This implies that patients in the high-risk category, displaying elevated EMT features, may experience less favorable outcomes from immunotherapy.

## Discussion

Intratumor heterogeneity is a pervasive and yet unresolved confounding factor in the discovery and application of biomarkers in cancer [16, 52, 53]. Consistent with reports in hepatocellular carcinoma, renal clear cell carcinoma, lung, and breast cancer, molecular biomarkers derived from a single biopsy may inaccurately represent a patient’s prognostic risk, either overestimating or underestimating it, when intratumor heterogeneity is not taken into account [10, 54–56]. In the realm of ESCC, multiregion sequencing analyses have revealed that existing ESCC models exhibit considerable discrepancies in risk assessment, ranging from 33% to 67%. This highlights the critical need for integrating multiregion sampling or spatial analysis and accounting for intratumor heterogeneity in the design of tumor biomarkers. In this study, we embarked on an analysis of the multiregion transcriptomic spectrum of ESCC, with the objective of identifying genes that exhibit low intratumor heterogeneity. We integrated multiple machine learning algorithms to develop a potent signature characterized by low intratumor heterogeneity. Consequently, we established an ESCC prognostic signature named ITHCS, comprising 39 genes. This signature maintained its generalizability and accuracy across the TCGA-ESCC and Zhang *et al*. cohorts.

Previous research has shown that multiregion tumor sequencing, by adequately accounting for intratumor heterogeneity, can contribute substantially to the construction of more refined prognostic models [9–11]. Furthermore, the selection of different regions within the same tumor for sequencing contributes to the stability of the results. In comparisons of the ITHCS with other models, ITHCS demonstrated superior predictive accuracy over 13 other signatures. Beyond multiregion bulk RNA-seq, where ITHCS displayed optimal stability in intratumor heterogeneity and maximal intertumor heterogeneity diversity, its advantages were equally evident at single-cell transcriptomic and spatial transcriptomic levels, underscoring its reliability at the micro level. Intriguingly, none of the 39 genes comprising ITHCS overlapped with the 96 genes reported in the past 13 models. Further investigation into these 39 genes in the context of ESCC revealed that except for CCND1, which is commonly amplified in ESCC and can act as an independent prognostic factor [57, 58], the remaining 38 genes were rarely reported in ESCC. Generally, when the impact of intratumor heterogeneity is overlooked, intertumor heterogeneity gets amplified with data extracted at various tumor levels. In this study, we adopted the approach of Biswas *et al*. [9] in lung cancer, constructing a heterogeneity quadrant chart comprising both intratumor and intertumor heterogeneity. All 39 genes in the model originated from the Q4 quadrant, maintaining high intertumor heterogeneity while ensuring stable intratumor heterogeneity.

Although TNM staging plays a crucial role in the diagnosis and treatment of ESCC patients, it currently fails to provide detailed risk stratification for early-stage ESCC patients, particularly those at Stage I. We further explored the association of ITHCS with the clinical features of ESCC patients. Given the limited number of Stage I patients in the GSE53625, TCGA-ESCC, and Zhang *et al*. datasets, we evaluated Stages I and II patients as early-stage ESCC and found that traditional TNM staging does not significantly differentiate the prognostic differences between Stages I and II, which is influenced by the cohort size. However, when assessed using ITHCS, it markedly distinguished early-stage ESCC patients. Compared to the other 13 models, ITHCS most distinctly differentiated the survival of early-stage ESCC patients.

Upon stratifying ESCC patients into high-risk and low-risk groups based on median values, we observed that genes upregulated in the high-risk ESCC group were significantly enriched in the EMT pathway. This enrichment might explain the poorer prognosis observed in high-risk patients. Intratumor heterogeneity manifests across multiple dimensions, including genomic, epigenomic, transcriptomic, and proteomic variations. Adhering to the central dogma of molecular biology, RNA serves as the executor of DNA, meaning that variations and modifications at the DNA level are expressed through RNA. Consequently, genomic heterogeneity and DNA epigenetic modifications are naturally reflected in RNA heterogeneity. Previous research has primarily focused on identifying the highest level of mutations and copy number variations in tumor evolution. However, we propose that transcriptomic heterogeneity might better reflect the biological phenotype and clinical pathological features of tumors. Therefore, biomarker design based on transcriptomic heterogeneity could be more direct, preferable, and superior to upstream omics heterogeneity, potentially circumventing the overall heterogeneity of tumors. While proteins, as the ultimate functional executors, also exhibit proteomic heterogeneity in tumors [59], proteomic measurement techniques are more expensive and complex than transcriptomic sequencing, leading to relatively scarce proteomic data and limited applications. Thus, ITHCS holds promise for using single-region ESCC samples to predict patient prognosis. Future studies should further explore biomarker design based on proteomic heterogeneity.

ITHCS has shown promising results, but further validation is needed before it can be translated into clinical practice. Future studies will focus on experimental validation, and developing a quantitative polymerase chain reaction panel based on the ITHCS gene signature could be highly beneficial. Furthermore, to effectively integrate ITHCS into clinical workflows and demonstrate its practical benefits, further validation studies and real-world research are necessary. We will conduct additional clinical trials and experiments, collecting more data from ESCC patients, to assess the tool’s practicality and potential for clinical decision-making in future studies. In summary, to our knowledge, this study is the first to combine multiregion RNA-seq datasets, single-cell transcriptomics, and spatial transcriptomics to explore intratumor heterogeneity in ESCC while circumventing this heterogeneity in the construction of an ESCC predictive model. By integrating multiple machine learning algorithms, we developed the prognostic signature ITHCS, capable of overcoming sampling bias. A higher ITHCS risk score is significantly associated with poor prognosis in ESCC patients and demonstrates exceptional efficacy in identifying early-stage patients. Additionally, the ITHCS risk score may correlate with tumor EMT characteristics. In conclusion, ITHCS offers more reliable prognostic insights for ESCC patients and may assist clinicians in selecting personalized treatment strategies. However, it is necessary to further refine the ITHCS prognostic model in prospective studies and large cohorts with multiregion ESCC samples.

**Key Points**

- The extensive intratumor heterogeneity in esophageal squamous cell carcinoma (ESCC) introduces significant sampling bias, undermining the reliability of prognostic models.
- The ITHCS predictive model is designed to minimize the effects of intratumor heterogeneity and circumvent the issues associated with sampling bias effectively.
- The ITHCS model demonstrates superior accuracy in prognostic monitoring for early-stage ESCC patients.

## Supplementary Material

Sup_Figures_bbae631

Supplementary_Tables_bbae631

## Data Availability

All sequencing data utilized in this study are freely accessible at the corresponding websites listed above. Additionally, the code for this study has been made publicly available at https://github.com/BiostarkTT/ITHCS. Further information can be reasonably requested from the corresponding authors.
